# Extract Derived from *Cedrus atlantica* Acts as an Antitumor Agent on Hepatocellular Carcinoma Growth In Vitro and In Vivo

**DOI:** 10.3390/molecules25204608

**Published:** 2020-10-10

**Authors:** Xiao-Fan Huang, Kai-Fu Chang, Shan-Chih Lee, Gwo-Tarng Sheu, Chia-Yu Li, Jun-Cheng Weng, Chih-Yen Hsiao, Nu-Man Tsai

**Affiliations:** 1 Institute of Medicine, Chung Shan Medical University, Taichung 40201, Taiwan; s9870509@gmail.com (X.-F.H.); kfchang1015@gmail.com (K.-F.C.); gtsheu@csmu.edu.tw (G.-T.S.); 2 Department of Medical Laboratory and Biotechnology, Chung Shan Medical University, Taichung 40201, Taiwan; 3 Department of Medical Imaging and Radiological Sciences, Chung Shan Medical University, Taichung 40201, Taiwan; sclee@csmu.edu.tw; 4 Department of Medical Imaging, Chung Shan Medical University Hospital, Taichung 40201, Taiwan; 5 Department of Life and Death, Nanhua University, Chiayi 62249, Taiwan; joyce@nhu.edu.tw; 6 Department of Medical Imaging and Radiological Sciences, Chang Gung University, Taoyuan 33303, Taiwan; jcweng@gmail.com; 7 Division of Nephrology, Department of Internal Medicine, Ditmanson Medical Foundation Chia-Yi Christian Hospital, Chiayi 60002, Taiwan; 8 Department of Hospital and Health Care Administration, Chia Nan University of Pharmacy and Science, Tainan 71710, Taiwan; 9 Clinical Laboratory, Chung Shan Medical University Hospital, Taichung 40201, Taiwan

**Keywords:** hepatocellular carcinoma (HCC), *Cedrus atlantica* extract (CAt extract), cell cycle, apoptosis

## Abstract

*Cedrus atlantica* is widely used in herbal medicine. However, the anti-cancer activity of *C. atlantica* extract (CAt extract) has not been clarified in hepatocellular carcinoma. In the study, we elucidated the anti-hepatoma capacity of CAt extract on HCC in vitro and in vivo. To explore the anti-hepatoma mechanisms of the CAt extract in vitro, HCC and normal cells were treated with the CAt extract, which showed marked inhibitory effects on HCC cells in a dose-dependent manner; in contrast, the CAt extract treatment was less cytotoxic to normal cells. In addition, our results indicate that the CAt extract induced apoptosis via caspase-dependent and independent apoptosis pathways. Furthermore, the CAt extract inhibited HCC tumor cell growth by restraining cell cycle progression, and it reduced the signaling of the AKT, ERK1/2, and p38 pathways. In the xenograft model, the CAt extract suppressed HCC tumor cell growth and prolonged lifespan by inhibiting PCNA protein expression, repressing part of the VEGF-induced autocrine pathway, and triggering strong expression of cleaved caspase-3, which contributed to cell apoptosis. Moreover, the CAt extract did not induce any obvious changes in pathological morphology or body weight, suggesting it had no toxicity. CAt extract exerted anti-tumor effects on HCC in vitro and in vivo. Thus, CAt extract could be used as a potential anti-cancer therapeutic agent against HCC.

## 1. Introduction

Hepatocellular carcinoma (HCC) is the most common type of liver cancer, with an estimated rank of fifth among cancer diagnoses and second among cancer deaths [1]. With the changes in lifestyle and diet patterns, the incidence and mortality rates of HCC are increasing worldwide [2]. In addition, the average survival rate of patients with HCC is less than three months, owing to the majority of patients being diagnosed in an advanced stage [3,4]. As a result, there are limited therapeutic options for patients, such as transcatheter arterial chemoembolization (TACE), combination chemo-drugs, and targeted therapy [5]. However, these palliative treatments suffer from several problems due to their toxicity and associated side effects [6]. Therefore, it is still urgent to develop a new anti-hepatoma agent for clinical use. 

For years, herbs and traditional Chinese medicine have been used in daily life and can be used as foods, flavoring agents, fragrances, or medicines that can alleviate discomfort and improve health. In addition, based on scientific research, various plant extracts have exhibited anti-tumor effects on many types of cancers [7]. Moreover, several studies have reported that plant extracts possess many biological functions, including anti-inflammation and anti-oxidation [8]. Currently, several clinical drugs are extracted from plants, such as etoposide, paclitaxel, irinotecan, and vincristine, which are applied to colon, breast, and lung cancer treatment [9]. Hence, natural plants might be an alternative and viable choice to treat HCC patients owing to their multitarget and coordinated intervention effects. 

The genus *Cedrus* contains at least four species: *C. deodara*, *C. libani*, *C. brevifolia*, and *C. atlantica*. Among these, *C. deodara* is widely studied in regard to different biofunctions, such as anti-inflammatory [10], anti-cancer [11,12,13,14,15,16], anti-oxidant [17], and antimicrobial activities [18,19]. In terms of its anti-cancer activity, it has been shown that *C. deodara* extract has obvious inhibitory effects on tumor growth in various cancers such as leukemia. However, the anti-tumor activity of *C. atlantica* has not been fully explored in vitro or in vivo on liver cancer. *C. atlantica* is an important forest tree species distributed in northern Africa, and its essential oil can be used as a flavoring agent in perfumery and cosmetology. It has been proposed as an antibacterial agent against Gram-positive bacteria [20], and it can alleviate pain by inhalation [21]. Hence, the aim of this study was to investigate the anti-tumor effects and mechanisms of *C. atlantica* extract (CAt extract) against HCC in vitro and in vivo. 

Our laboratory described the significance of the CAt extract on the suppression of HCC tumor growth in vitro and in vivo. Furthermore, we extended our understanding of its anti-cancer mechanisms to the alteration of cell cycle progression, the mediation of cell apoptosis, and the inhibition of cell proliferation. Then, its histopathology was further evaluated. Consequently, our new understanding of the mechanisms of action of the CAt extract suggest a potential for CAt extract to be developed as an anticancer agent for HCC therapy.

## 2. Results

### 2.1. CAt Extract Inhibited the Cell Growth of HCC Cells In Vitro

**Figure 1:**
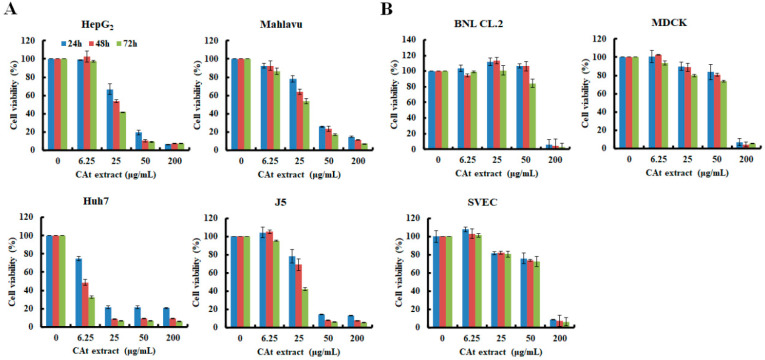
Effects of the *Cedrus atlantica* extract (CAt extract) extract on the growth inhibition of hepatocellular carcinoma (HCC) (**A**) and normal (**B**) cells. Cells were cultured in 96-well plates overnight and treated with or without CAt extract (0–200 μg/mL) for 24, 48, and 72 h, and evaluated by an MTT assay. Data are expressed as the mean ± SD from two independent experiments.

In exploring the potential inhibitory effects of CAt extract against HCC cells, which were treated with a serial dilution of the CAt extract for 24, 48, and 72 h, the results revealed that CAt extract effectively repressed 50% of HCC cell growth at the concentration of 25 μg/mL (Figure 1A). Among the HCC cell lines assessed, Huh7 cells were the most sensitive, with an 80% decline of viability at 25 μg/mL. In the others, their viability drastically dropped to 20% at a concentration of 50 μg/mL. These results reveal that the viability of HCC cells was reduced in a time- and dose-dependent manner after CAt extract treatment. Moreover, the cytotoxicity of the CAt extract was examined for normal cells, including BNL CL.2 (mouse normal liver embryonic cells), MDCK (canine normal kidney epithelial cells), and SVEC (mouse normal endothelial cells), and the results show that the CAt extract did not apparently affect cell viability below 50 μg/mL at different time points (Figure 1B). Then, as shown in Table 1, the IC_50_ values of the CAt extract on HepG_2_, Mahlavu, J5, and Huh7 cells were 27.09 ± 1.83 μg/mL, 33.57 ± 2.84 μg/mL, 32.83 ± 4.31 μg/mL, and 6.09 ± 3.28 μg/mL at 24 h. The 50% inhibitory concentrations of the CAt extract on normal cells, respectively SVEC, MDCK, and BNL CL.2, were 68.03 ± 4.05 μg/mL, 69.98 ± 1.56 μg/mL, and 150.03 ± 9.57 μg/mL. In summary, the CAt extract effectively inhibited HCC cell growth without markedly being cytotoxic to normal cells. 

### 2.2. CAt Extract Blocked Cell Cycle Progression at G_0_/G_1_ Phase of the HepG_2_ and Mahlavu Cells

**Figure 2:**
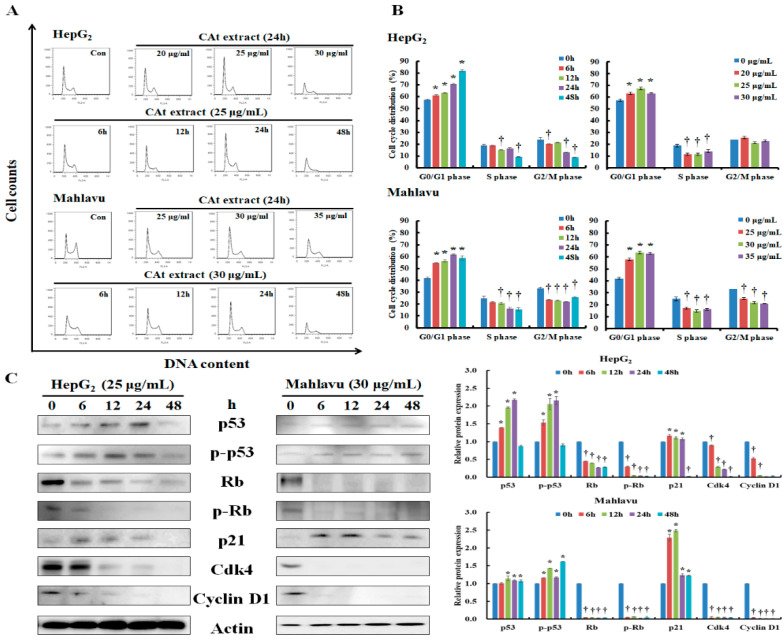
Effects of CAt extract treatment on the cell cycle distribution and cell cycle regulators expression in HepG_2_ and Mahlavu cells. Cells were treated with the indicated CAt extract concentrations for 6, 12, 24, and 48 h. After harvesting, the cells were stained with propidium iodide (PI) and analyzed by flow cytometry. Representative diagram of cell cycle progression (**A**). Representative histograms of cell cycle distribution (**B**). The protein expression levels of the cell cycle regulators were assessed by Western blot (**C**). The actin level was used as loading control. *, †: Significant difference between control and treatment, *p* < 0.05.

Former data indicated that CAt extract showed inhibitory effects on the HepG_2_ with wildtype p53 and Mahlavu with mutant p53 (C278L) cells [22,23], and therefore the cell cycle progression was further analyzed. A change of cell cycle was detectable after CAt extract treatment, and the CAt extract induced cell cycle arrest at G_0_/G_1_ phase in both cell lines (Figure 2A). Moreover, the CAt extract impeded the cell cycle of HepG_2_ cells by increasing the population in G_0_/G_1_ phase (57.35 ± 0.86%, 61.22 ± 0.61%, 63.13 ± 0.14%, 70.45 ± 0.62%, and 81.85 ± 0.81%) and reducing S phase (18.7 ± 1.25%, 18.75 ± 0.5%, 15.38 ± 0.10%, 16.38 ± 0.59%, and 9.33 ± 0.46%) as well as G_2_/M phase (23.90 ± 1.77%, 20.03 ± 0.32%, 21.49 ± 0.06%, 13.17 ± 0.14%, and 8.82 ± 0.42%) in a time-dependent manner. Treating HepG_2_ cells with different concentrations of the CAt extract showed that the CAt extract also induced cell cycle arrest in G_0_/G_1_ phase and reduced S phase. The cell cycle distribution of the Mahlavu cells was markedly arrested at G_0_/G_1_ phase (41.94 ± 0.78%, 54.71 ± 0.19%, 56.42 ± 1.01%, 61.84 ± 0.66%, and 58.76 ± 1.89%) and there was a significant time-dependent decrease of the S phase (24.83 ± 1.78%, 21.73 ± 0.64%, 20.6 ± 0.81%, 16.32 ± 0.81%, and 15.44 ± 1.2%) and G_2_/M phase (33.22 ± 1.07%, 23.55 ± 0.45%, 22.94 ± 0.51%, 21.83 ± 0.49%, and 25.79 ± 0.61%) after CAt extract treatment (Figure 2B). Additionally, treating Mahlavu cells with different concentrations of CAt extract presented a similar trend to accumulate the cell population at G_0_/G_1_ phase followed by decreasing both S and G_2_/M phases. These results reveal that the CAt extract impeded cell cycle progression at G_0_/G_1_ phase in both HepG_2_ and Mahlavu cells. Next, the mechanisms of CAt extract’s regulation of cell cycle in the HepG_2_ and Mahlavu cells was examined. As reported in the literature, p53 can induce p21 activation and mediate cyclin/CDK complex degradation (e.g., cdk4 and cyclin D1), resulting in cell cycle arrest. As Figure 2C shows, after CAt extract treatment, HepG_2_ cells with wild-type p53 dramatically increased p53 and p-p53 (Ser392) expression at 6 h, sustained to 24 h, and diminished at 48 h. Afterward, the level of p21 was also suddenly increased at 6 h and gradually decreased, and the level of cdk4 and cyclin D1 rapidly decreased from 6 h to 48 h. These results reveal that the CAt extract rapidly activated p53 and p21, resulting in the downregulation of cdk4 and cyclin D1 in HepG_2_ cells. Rb is a tumor suppressor that can alter cell cycle progression to affect cell proliferation. The results indicate that CAt extract significantly reduced the level of Rb and p-Rb (Ser24/Thr252) in HepG_2_ cells. Further, Mahlavu cells with mutant p53 were observed to have induced p53 and p-p53 (Ser392) expressions, which maintained the protein expression levels from 6 h to 48 h and the level of p21 was abundantly increased at 6 and 12 h, resulting in cdk4 and cyclin D1 expressions being sharply diminished at 6 h after CAt extraction treatment. These results reveal that CAt extract rapidly increased p21 expression and sustained the increase of activated p53 expression to 48 h to downregulate cdk4 and cyclin D1 quickly. The level of Rb and p-Rb (Ser24/Thr252) was quickly reduced in Mahlavu cells after CAt extract, revealing that CAt extract repressed Rb and p-Rb expressions were stronger than in HepG_2_ cells. Consequently, the CAt extract increased p53 and p-p53 (Ser392) and affected downstream protein expressions, including p21, cdk4, and cyclin D1, to affect cell cycle progression. CAt extract also moderated Rb and p-Rb (Ser24/Thr252) protein expressions to mediate cell cycle progression in HepG_2_ and Mahlavu cells.

### 2.3. CAt Extract Repressed AKT, ERK, and p38 Protein Expression in HepG_2_ and Mahlavu Cells

**Figure 3:**
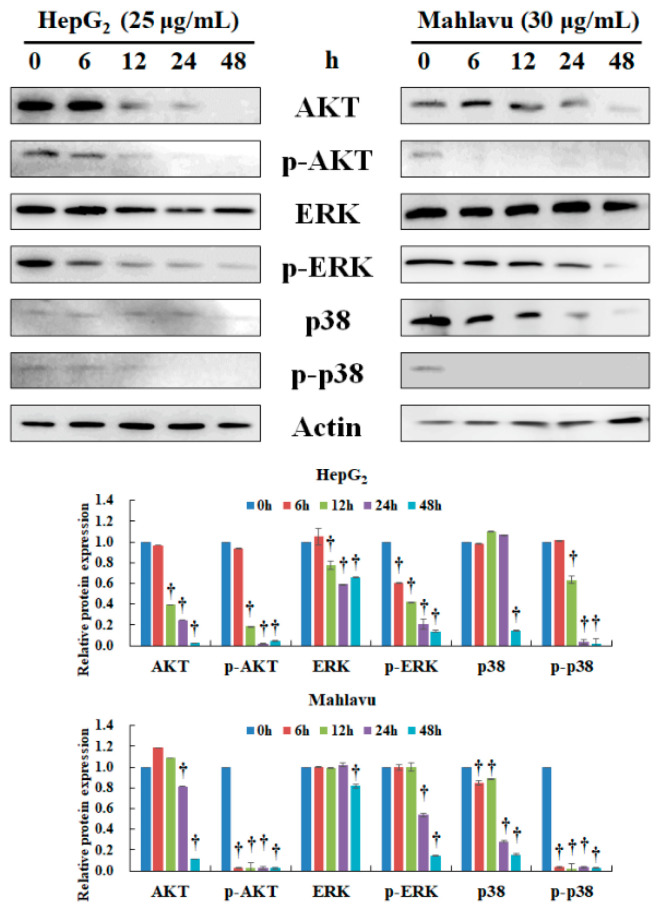
Effect of CAt extract treatment on AKT, ERK, and p38 protein expression in HepG_2_ and Mahlavu cells. HepG_2_ and Mahlavu cells were incubated with CAt extract (25 or 30 μg/mL) for the indicated time points (0, 6, 12, 24, and 48 h) then subjected to Western blotting analysis. The actin level was used as loading control. †: Significant difference between control and treatment, *p* < 0.05.

The biologically relevant signaling pathways of hepatocarcinogenesis involve the AKT, ERK, and p38 pathways [24,25], and thus the expression of these proteins was elucidated by Western blot. After CAt extract treatment, AKT protein expression was significantly reduced, and there was also inhibition of the phosphorylation of p-AKT (Ser473/Ser474/Ser472) at 12 h in HepG_2_ cells (Figure 3). The protein expression of ERK was decreased at 12 h, and the level of p-ERK (Tyr204) rapidly reduced at 6 h, which continuously declined to 48 h in a time-dependent manner in HepG_2_ cells. Further, the level of p-p38 (Tyr182) was decreased by CAt extract and showed a similar trend with the inhibition of p-AKT protein expression in HepG_2_ cells. In Mahlavu cells, the CAt extract significantly reduced AKT protein expression at 24 h and remarkably diminished the level of p-AKT (Ser473/Ser474/Ser472) at 6 h. However, the level of ERK and p-ERK (Tyr204) were not rapidly inhibited by CAt extract, and these protein expressions were significantly reduced at 48 h. Next, the level of p38 was reduced at 6 h, lasting to 48 h, and p-38 (Tyr182) was suddenly declined at 6 h in Mahlavu cells. Consequently, the CAt extract displayed good inhibitory ability through the AKT/p-AKT, ERK/p-ERK, and p38/p-p38 pathways, which facilitate HCC cell growth. 

### 2.4. CAt Extract Induced Cell Apoptosis of HCC Cells through Activation of the Extrinsic and Intrinsic Caspase Cascades

**Figure 4:**
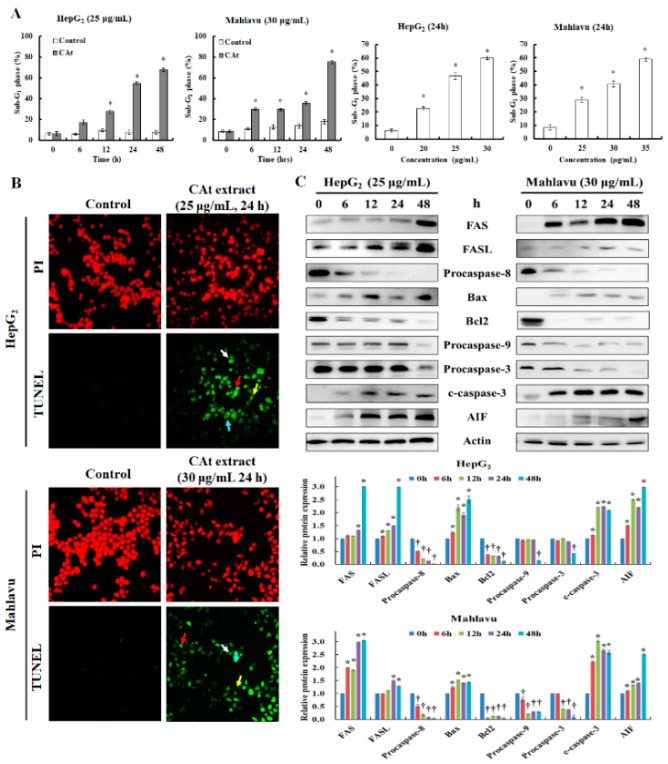
Effects of CAt extract treatment on apoptosis in HepG_2_ and Mahlavu cells. The percentage of sub-G_1_ phase which was analyzed by flow cytometry after CAt extract treatment with HepG_2_ and Mahlavu cells (**A**). Photograph of TUNEL expression on HepG_2_ and Mahlavu cells treated with or without CAt extract (25 or 30 μg/mL) for 24 h by fluorescence microscope (**B**), red arrow: anoikis; blue arrow: chromatin condensation; yellow arrow: DNA fragments; white arrow: apoptotic bodies. Cells treated with CAt extract (25 or 30 μg/mL) for 0, 6, 12, 24, and 48 h, and cell lysates were collected for analysis of indicated protein expression. Effects of CAt-extract-induced apoptotic protein expression was analyzed by Western blot (**C**). c-caspase-3: cleaved caspase-3. *, †: Significant difference between control and treatment, *p* < 0.05.

Due to the growth-inhibitory effect of CAt extract on HCC cells, the sub-G_1_ phase was further examined to detect whether CAt extract induced cell death. The results reveal that sub-G_1_ phase significantly increased to about 60–80% of sub-G_1_ phase in both cells in a time- and dose-dependent manner (Figure 4A). Then, the mode and mechanism of CAt extract’s induction of apoptotic cell death in HepG_2_ and Mahlavu cells were further examined. In the TUNEL assay results, the cell nucleus of the untreated HepG_2_ and Mahlavu cells with PI staining were round and flat, and no distinct green fluorescence of TUNEL was observed. However, after treatment with the CAt extract, the nuclei were fragmented into different sizes in most of the cells, and the green fluorescence of TUNEL was strongly visible along with various apoptotic cell morphologies, including anoikis, DNA fragments, chromatin condensation, and apoptotic bodies formation (Figure 4B). Consequently, to investigate the CAt-extract-induced apoptotic mechanisms in the HepG_2_ and Mahlavu cells, the apoptosis-associated protein expression levels were evaluated (Figure 4C). The extrinsic apoptosis-related proteins, including FAS, FASL, and procaspase-8, were examined, and the results indicate that the FAS and FASL proteins were upregulated in treated Mahlavu and HepG_2_ cells. The downstream pro-caspase-8 protein expression was markedly decreased at 6 h and kept reducing to 48 h, revealing that the extrinsic pathway was activated. The intrinsic apoptotic proteins were also investigated, and the results show that an increment of Bax and a decline of Bcl2 and procaspase-9 were significantly reduced at 48 h in HepG_2_ cells, indicating that the intrinsic pathway was turned on. Increasing levels of procaspase-3 and decreasing levels of cleaved caspase-3 were observed at 48 h, suggesting that the caspase cascade was fully activated at 48 h in HepG_2_ cells. Besides, the level of AIF (apoptosis-inducing factor) was dramatically increased at 6 h, and that continuously amplified its protein expression in HepG_2_ cells. Moreover, the levels of FAS and FASL proteins were sharply increased, followed by a decline of procaspase-8 at 6 h to the activated extrinsic pathway in Mahlavu cells. The intrinsic apoptotic proteins, including Bax, Bcl2, and procaspase-9, were rapidly affected by the CAt extract at 6 h. The levels of Bax and Bcl2 showed a similar trend to what was detected in HepG_2_ cells, and the downstream protein procaspase-9 was decreased at 6 h to facilitate activation of the intrinsic pathway in Mahlavu cells. In addition, procaspase-3 protein expression was reduced, and cleaved caspase-3 protein expression was increased in a time-dependent manner in Mahlavu cells. The level of AIF in Mahlavu cells was increased after CAt extract treatment, resulting in activation of the caspase-independent pathway. Therefore, the CAt extract blocked cell proliferation via the induction of cell apoptosis, including the extrinsic as well as the intrinsic caspase-dependent and independent apoptosis pathways.

### 2.5. CAt Extract Suppressed HCC Xenograft Tumor Growth and Extended Lifespan

**Figure 5:**
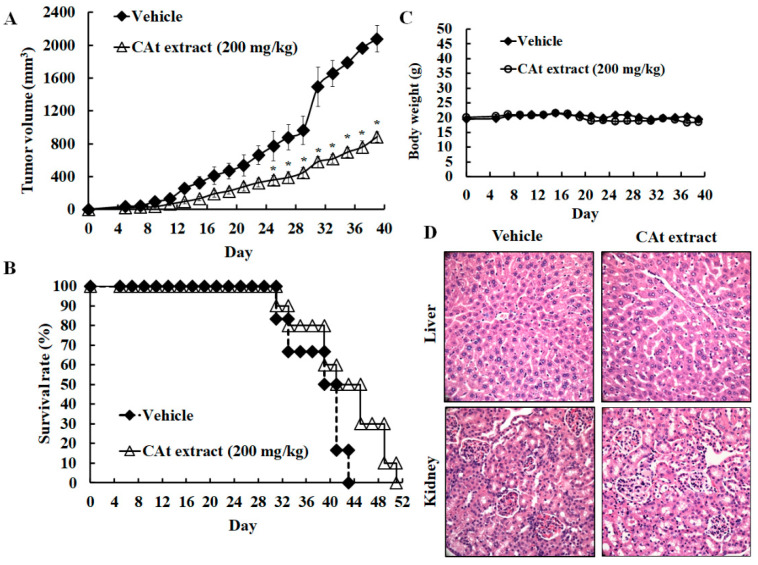
The xenograft tumor model of HCC treated with the CAt extract (200 mg/kg). The tumor suppression (**A**) and the survival rate (**B**) of HCC tumor growth after CAt extract treatment. Evaluation of systemic toxicity of CAt extract administration on the HCC xenograft. The body weight after CAt extract treatment (**C**). The hematoxylin/eosin (H&E) staining of the liver and kidney (**D**).

To examine the inhibitory potential of the CAt extract on HCC tumors, an HCC xenograft model was established. Mice bearing HepG_2_ liver cancer xenografts were subcutaneously injected with mineral oil as a vehicle or treated with the CAt extract (200 mg/kg, s.c.) every two days. When the tumor volume was greater than 2000 mm^3^, the mice were sacrificed to collect the tumor as well as the organs for follow-up pathological analysis. The data reveal that the CAt extract treatment achieved about a 42.3% inhibitory effect at day 39 on the tumor burden of the HCC xenograft compared with the vehicle (Figure 5A; 882.4 ± 62.9 mm^3^ versus 2077.2 ± 160.2 mm^3^). The data also demonstrate that the CAt extracts prolonged the lifespan from 43 to 51 days compared with the vehicle (Figure 5B). Thus, these data demonstrate that the CAt extract has antitumor effects on HCC tumor growth and improves survival outcomes in vivo. Subsequently, to evaluate the CAt-extract-induced systematic toxicity in vivo, the body weights of the mice were measured every two days. The results revealed no significant difference between the CAt extract treatment and vehicle in changes of body weight, suggesting that the CAt extract might have little toxicity in vivo (Figure 5C). In addition, a pathological evaluation of hematoxylin/eosin (H&E)-stained liver and kidney tissues was conducted; there were no obvious changes in tissue morphology (Figure 5D). The results demonstrate that the CAt extract might show little or no toxicity as assessed by body weight and organ damage.

### 2.6. CAt Extract Exhibited Antihepatoma Capacity via Induction of Apoptosis, Reduction of Cell Proliferation, and a Decline of Metastatic Protein Markers

**Figure 6:**
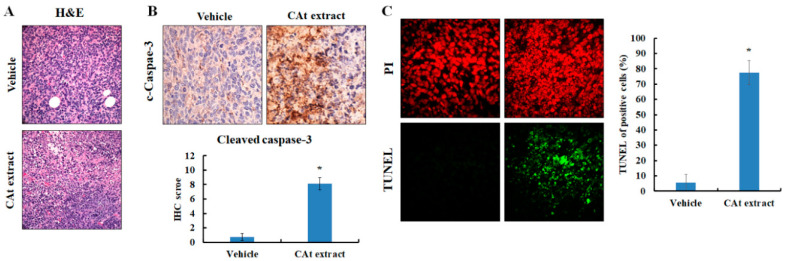
CAt-extract-induced apoptosis in the HCC xenograft tumor model by H&E, immunohistochemical (IHC), and TUNEL staining. (**A**) Tissue morphology, evaluated with H&E staining. (**B**) CAt-extract-induced cleaved caspase-3 expression. (**C**) TUNEL staining for the observation of apoptotic cells. *: A significant difference between control and treatment, *p* < 0.05.

**Figure 7:**
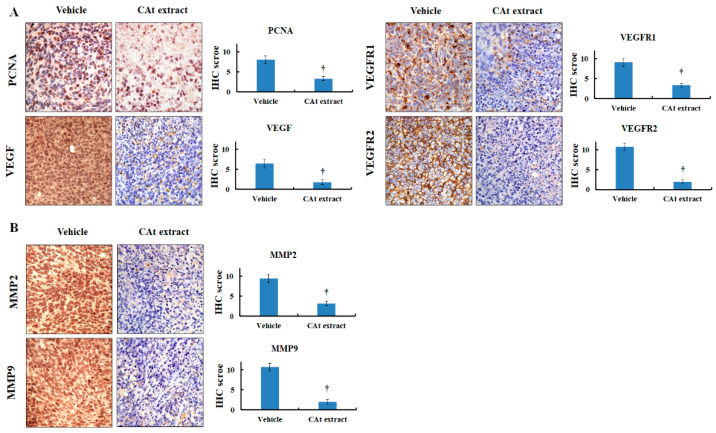
CAt-extract-induced inhibitory effect in the HCC xenograft tumor model by IHC staining. Indicated protein expression in HCC tumor tissues was stained by IHC. (**A**) The level of PCNA, VEGF, VEGFR1, and VEGFR2 protein. (**B**) The level of MMP2 and MMP9 protein. †: A significant difference between control and treatment, *p* < 0.05.

Next, to further elucidate the mechanisms of the CAt extract’s effects on the HCC xenograft animal model, the CAt-extract-induced tumor cell damage was observed by H&E staining, and the results reveal that the CAt extract triggered tumor cell death (Figure 6A). Thus, we looked for evidence of cell apoptosis induced by the CAt extract. The results show that the protein expression of cleaved caspase-3 was increased and that apoptotic bodies were observed in the tumor tissue (Figure 6B). Moreover, the TUNEL assay revealed that many DNA fragments were observed in many of the hepatoma cells, and the CAt extract induced a great number of TUNEL-positive cells (Figure 6C). These results reveal that the CAt extract triggered tumor cell death by inducing cell apoptosis. Then, in order to identify the inhibitory mechanisms of the CAt extract in HCC xenografts, the protein expression of PCNA, VEGF, VEGFR1, and VEGFR2 were examined by immunohistochemical (IHC) staining. PCNA is expressed during the DNA synthesis phase of the cell cycle. The CAt extract reduced the expression of the PCNA protein, suggesting that it might possess the ability to affect cell cycle progression in hepatoma cells (Figure 7A). VEGF and its target receptors, VEGFR1 and VEGFR2, were examined in order to investigate the inhibitory effects of autocrine VEGF signaling, which might be affected by the CAt extract. The results indicate that VEGF, VEGFR1, and VEGFR2 protein expressions were all reduced by the CAt extract treatment, suggesting the CAt extract might reduce VEGF autocrine signaling. Moreover, MMP2 and MMP9 protein expression were tested in order to investigate the anti-metastasis potential of the CAt extract, and we found that the CAt extract repressed the expression of both MMP2 and MMP9, suggesting that the CAt extract might have the potential to suppress HCC invasion by inhibiting MMP2 and MMP9 protein expression (Figure 7B). Taken together, the CAt extract might have several anti-HCC effects in vivo, with results that are consistent with the data found in vitro, including the induction of apoptosis and interruption of cell proliferation. Consequently, we aimed to analyze the effective components in CAt extract by GC-MS, and as shown in Table 2, the five major components were thujopsene (43.36%), α-cedrene (31.67%), α-cadinene (2.73%), cedrol (1.42%), and isolongipholene (0.52%). Cedrol has reported anti-cancer activity, [26] and might be an effective compound to inhibit HCC cell growth. 

## 3. Discussion

Herbal and plant medicines have been used for centuries worldwide. Furthermore, in recent decades, a significant number of medicinal herbs have been reported to have biofunctional effects in treating chronic liver diseases through eliminating viruses, alleviating fibrogenesis, and suppressing tumorigenesis. As a result, plant extracts can be considered as potential anti-cancer candidates for cancer therapy [9,10]. Here, in the first study to investigate the anti-hepatoma activity of CAt extract in vitro and in vivo, our results first found that HCC cells were inhibited by CAt extract; nevertheless, the cytotoxicity of CAt extract was moderate against normal cells, including liver embryonic cells, kidney epithelial cells, and endothelial cells. The results also indicate that CAt extract blocked cell cycle progression at G_0_/G_1_ phase in vitro. In addition, p53 protein prominently increased from 6 to 24 h, and the functional phosphorylated p53 was increased by CAt extract. The downstream protein, p21, was increased after the increment of p53 and p-p53 protein expression, and cdk4 and cyclin D1 were found to be reduced in a time-dependent fashion. The alteration of the cell-cycle-related protein expression levels contributed to stopping cell cycle progression. Besides, TP53 mutation is associated with a poor prognosis [27]. Our result indicates that CAt extract exerted an inhibitory effect on both wild-type and mutant p53 HCC cells. Moreover, total Rb and phosphorylated Rb were dramatically decreased within 6 h, which might increase the dissociation rate of E2F and Rb to alleviate cell proliferation. On the other hand, MAPK and AKT/mTOR signaling pathways are essential in controlling cell proliferation, differentiation, and survival, and have been studied to determine the pathogenesis of HCC [24,25]. The AKT/mTOR pathway plays a pivotal role in hepatocarcinogenesis [28,29], and a previous study showed about 53% positive expression of p-AKT protein in HCC tissues and 12% of cirrhotic tissues [30]. Therefore, it is an attractive candidate as an anticancer drug target for HCC treatment. Moreover, ERK can regulate cell cycle progression, apoptosis resistance, cellular motility, and drug resistance. As a result, a repression of ERK was exerted to inhibit the development of HCC and increase cell apoptosis in HCC tumors [31]. We first found that phosphorylated AKT, ERK, and p38 were reduced rapidly within 12 h after CAt extract treatment, suggesting that CAt extract has a potential capacity to suppress hepatocarcinogenesis, helping to prevent the development of HCC at an early stage. 

Next, we used TUNEL staining to test whether the CAt extract induced cell apoptosis, and found that many cells were TUNEL green positive. The details of the mechanism of apoptosis induction were examined. After CAt extract treatment, FAS and FASL expression increased at 12–24 h, which might have caused the expression of the procaspase-8 protein to decrease at 12 h dramatically. However, procaspase-8 decreased before FAS and FASL protein increased, which suggests that the CAt extract might regulate the extrinsic apoptotic pathway via different mechanisms, which we explored further. CAt extract might not directly affect the intrinsic apoptotic pathway, as Bax, Bcl2, and procaspase-9 proteins did not show rapid declines in HepG_2_ cells. In the Mahlavu cells, the CAt extract rapidly increased Bax protein expression and sharply decreased Bcl2 and procaspase-9 protein expression at 6 h. In the late stage of apoptosis, CAt extract strongly and quickly induced cleaved caspase-3 protein expression in both cell lines. These results reveal that CAt extract triggered cell apoptosis via extrinsic as well as intrinsic apoptosis pathways. The alteration of AIF protein expression was greatly increased at 12 to 24 h, suggesting that CAt extract induced not only the caspase-dependent pathway but also activated the caspase-independent pathway, which contributed to cell apoptosis. Taken together, the results suggest that CAt extract suppressed HCC tumor growth via the inhibition of cell cycle progression, induction of cell apoptosis, and suppression of AKT, ERK, and p38 signaling in vitro. 

In the xenograft model, the results first demonstrate that CAt extract exerted a good HCC tumor-suppression ability. As seen by H&E staining, a marked anaphase cell morphology of the HCC tissue was observed. Many of the cell bodies were shrinking and the cell nuclei were fragmenting, resulting in cell death. Similarly, HCC tumor tissue stained for TUNEL revealed that the CAt extract induced cell apoptosis, with the formation of apoptotic bodies and DNA fragmentation. In addition, cleaved caspase-3 was strongly expressed, confirming that CAt extract induced cell apoptosis of HCC in vitro. Former results revealed that CAt extract regulated cell-cycle-related proteins to impede cell cycle progression. PCNA is a proliferation marker and can stand for the status of cell cycle. Moreover, HCC cells highly express PCNA, which increases during late G_1_ or very early S phases of the cell cycle [32]. As a result, in an in vivo study, PCNA was assayed in order to evaluate whether CAt extract affected cell cycle progression. CAt extract efficiently reduced PCNA protein expression, indicating that it inhibited HCC tumor cell growth, consistent with the results observed in vitro. Subsequently, we wondered whether CAt extract repressed VEGF/VEGFR-induced autocrine or paracrine proliferation in vivo; therefore, VEGF, VEGFR1, and VEGFR2 expressions were examined. In addition, VEGF/VEGFR signaling not only plays a critical role in the angiogenesis of HCC, but also has a vital role in the autocrine progression of HCC [33,34]. Previous studies have reported that VEGFR1 and VEGFR2 are overexpressed in HCC, and high levels of VEGF, VEGFR1, and VEGFR2 indicate a poor prognosis [35,36]. The results indicate that CAt extract exerted an inhibitory potential in the suppression of VEGF/VEGFR-induced autocrine or paracrine proliferation by downregulating VEGF, VEGFR1 and VEGFR2 expressions in vivo. Hence, these results provide a different and potential inhibitory effect of CAt extract by the restriction of the VEGF/VEGFR autocrine growth pathway to suppress HCC tumor growth and improve the prognosis of HCC patients. Additionally, some studies have indicated that the activated PI3K/AKT signaling pathway is correlated with hepatic cancer progression and promotes VEGF/VEGFR1 expression [37,38,39]. Therefore, the results might also suggest that CAt extract not only inhibited the activated AKT expression of upstream protein in vitro but also repressed VEGF, VEGFR1, and VEGFR2 expressions in vivo to suppress HCC tumor cell growth. Furthermore, CAt extract reduced MMP2 and MMP9 protein expression, which play essential roles in the invasion, revealing that CAt extract might possess anti-invasion activity in vivo. Hence, CAt extract was observed to have multiple functions in this study: growth inhibition, reduction of autocrine signaling, induction of apoptosis, and suppression of metastasis. To further find the antihepatoma compound in CAt extract, our results reveal that the cedrol might be one of the effective anticancer ingredients in CAt extract due to its anticancer activity in non-small-cell lung cancer [26], and others might need to conduct further study to explore the anticancer activity in future. 

During hepatocarcinogenesis, the liver function is damaged due to virus attack, alcohol abuse, or the accumulation of fatty acids. Therefore, liver toxicity is an important criterion for clinicians to evaluate the feasibility of treating HCC patients. We thus evaluated the changes of body weights and the pathological alterations of liver and kidney tissue after treatment with CAt extract. The results reveal that the CAt extract had almost no effect on body weight during the whole course of treatment. The liver tissue showed healthy liver cells with the filling of the cytoplasm, a complete cell membrane, and a nucleus. In the kidney tissue, the integrity of the glomerulus was intact. CAt extract treatment did not induce any obvious pathological morphology. Taken together, CAt extract had little toxicity towards organs, and these data confirm the in vitro cytotoxicity evaluation, suggesting that CAt extract might have few side effects in a human clinical trial. 

Taken together, the results show that CAt extract had an inhibitory effect on HCC tumor cell growth through the induction of apoptosis, the arrest of the cell cycle, the repression of autocrine signaling, and the inhibition of AKT/ERK/p38 signaling. Moreover, CAt extract has a potential capacity for the suppression of invasion. The evaluation of toxicity in the xenograft model revealed no or few changes in the mice. As a result, our findings provide a basis for further studies of CAt extract in the treatment of HCC. However, additional investigation is needed to clarify the molecular pathway precisely. Overall, it can be said that CAt extract has the potential for clinical applications in vitro and in vivo against HCC. 

## 4. Materials and Methods

### 4.1. Cell Culture

Human hepatocellular carcinoma cells (HepG_2_, Mahlavu, Huh7 and J5), mouse normal liver embryonic cells (BNL CL.2), mouse normal endothelial cells (SVEC), and canine normal kidney epithelial cells (MDCK) were purchased from the American Type Culture Collection (Manassas, VA, USA) and the Bioresource Collection and Research Center (Hsinchu, Taiwan). The cells were grown in Dulbecco′s modified Eagle′s medium (HepG_2_, Mahlavu, Huh7, BNL CL.2, SVEC, and MDCK cells) or Roswell Park Memorial Institute 1640 (J5 cell) supplemented with 10% fetal bovine serum, 1% sodium pyruvate, 1% HEPES, and 1% penicillin/streptomycin in an incubator containing 5% CO_2_ at 37 °C. All reagents were purchased from Gibco (Waltham, MA, USA.). The status of the genes in the cells was determined by the FemtoPath Primer Set (HongJing Biotech, New Taipei City, Taiwan). 

### 4.2. Extraction of Cedrus atlantica Plant Material 

The steam-distilled preparation of *Cedrus atlantica* extract (CAt extract) was purchased from PHOENIX (Red Bank, NJ, USA). For small-scale extraction, fresh bark of a *Cedrus atlantica* plant (500 g) was used, which was originally from America. The flow rate of generated steam was 7.2 mL/min, passed through the plant material at 100~105 °C for 100 min. After extraction, two layers were formed: one was the aqueous layer, and the other was the lipid layer utilized throughout the experiments. The extracted CAt solution (lipid layer) was aliquoted and sealed in a brown glass bottle and then stored at 4 °C. The CAt extract concentration was calculated in μg/mL and was diluted in DMSO (Sigma Aldrich, Saint Louis, MI, USA) such that the final concentration of solvent in culture media did not exceed 0.2%. 

### 4.3. Cytotoxicity of the CAt Extract on HCC Cells

HCC cells (5 × 10^3^ cells/well), BNL CL.2 (4 × 10^4^ cells/well), MDCK, and SVEC (1 × 10^4^ cells/well) were seeded and grown in 96-well plates overnight. Cells were treated with vehicle (0.1% DMSO) and serial concentrations of the CAt extract (0–200 μg/mL) for 24, 48, and 72 h. Cell viability was measured by MTT-based colorimetric assays. The formazan crystals formed were solubilized by DMSO, and the absorbance was measured at a wavelength of 550 nm by a SpectraMax M5 microplate reader (Molecular Devices, San Jose, CA, USA). The effective absorbance was calculated for cell viability (%), which was presented as treatment (OD)/vehicle (OD) × 100%. The experiments were analyzed in triplicate. 

### 4.4. Flow Cytometric Analysis

HepG_2_ and Mahlavu cells were plated in 10 cm dishes at a density of 2 × 10^6^, incubated overnight, and treated for 6, 12, 24, or 48 h with the indicated concentration of the CAt extract. The cells were trypsinized, washed with PBS, and resuspended in PBS containing 40 μg/mL propidium iodide (PI, Sigma Aldrich, Saint Louis, MO, USA) and 100 μg/mL of RNase A (Sigma Aldrich, Saint Louis, MO, USA) overnight in the dark. The cell cycle progression was detected by a FACS Calibur instrument (BD, Franklin Lakes, NJ, USA), and the percentage of cell cycle distribution was analyzed by FlowJo 7.6.1 (Ashland, OR, USA). Values given are the mean ± SD of three different experiments performed in triplicate. 

### 4.5. TUNEL Assay 

HepG_2_ and Mahlavu cells (2 × 10^5^) were seeded in 6-well plates overnight. The cells were treated with vehicle or the CAt extract (25 or 30 μg/mL) for 24 h. The cells were fixed using 4% formaldehyde/methanol at 4 °C for 10 min on slides and stained with terminal deoxynucleotidyl transferase (TdT) according to the manufacturer′s instructions (In Situ Cell Death Detection Kit, Roche, Basel, Switzerland). After PI staining (10 μg/mL), the slides were sealed with a cover glass. Each experiment was carried out in triplicate. The fluorescence was visualized by microscopy (ZEISS Axio Imager A2, Bremen, Germany) under ×400 field and the images were adjusted in Photoshop (Adobe, San Jose, CA, USA). 

### 4.6. Western Blot Analysis

HepG_2_ and Mahlavu cells (2 × 10^6^) were grown in 10 cm dishes overnight and treated with the CAt extract at different time points with the indicated CAt extract concentration. The protein concentration was measured by the BCA Protein Assay Reagent (Thermo Fisher Scientific, Waltham, MA, USA), according to the manufacturer’s instructions. The proteins were resolved by 8–12% SDS-PAGE and transferred to PVDF membranes. After blocking with skimmed milk, the membranes were incubated with primary antibodies overnight at 4 °C, then incubated with secondary antibodies conjugated to HRP (horseradish peroxidase) for 2 h at room temperature. Enhanced chemiluminescence detection reagents (T-Pro Biotechnology, New Taipei City, Taiwan) were used to visualize the protein expression, and the images were analyzed by a LAS-4000 Luminescence/Fluorescence Imaging System (GE Healthcare, Uppsala, Sweden). The protein expression was quantified using ImageJ software (NIH, Bethesda, MD, USA). All of the primary and secondary antibodies were purchased from Santa Cruz Biotechnology, Inc. (Dallas, TX, USA) and iReal Biotechnology Co., Ltd. (Hsinchu, Taiwan). All analyses were performed in biological duplicates. 

### 4.7. HCC Xenograft Model

Female BALB/c nude mice (6–8 weeks old) were obtained from the National Laboratory Animal Center, Taipei, Taiwan. All animal work was conducted in accordance with the protocol approved by the Laboratory Animal Center of Chung Shan Medical University (Taichung, Taiwan; no. CSMU-IACUC-1662). HepG_2_ cells (1 × 10^6^/100 μL/mouse) were subcutaneously injected into the right-backs of the mice, and drug administration was initiated five days later. All mice with xenografts were randomly separated into two groups: one was vehicle-treated with mineral oil, 100 μL (*n* = 6), and the other was treated with 200 mg/kg/100 μL CAt extract (*n* = 10). Treatments were given every two days by subcutaneous injection and, meanwhile, the tumor volume and the body weight were recorded. The tumor volume (TV) was calculated using the formula: length × width × height. All of the mice were sacrificed when the tumor volume reached 2000 mm^3^ as the last survival day. After sacrifice, the tumors and organs (heart, liver, spleen, lung, kidney, stomach, and intestine) were collected. The tissues were fixed with 10% neutral formalin and embedded in paraffin for immunohistochemical and H&E staining. The photographs were taken at ×400 by microscopy (ZEISS Axio Imager A2, Bremen, Germany).

### 4.8. Immunohistochemistry (IHC) and Hematoxylin/Eosin (H&E) Staining 

Excised tumors and organs were fixed in 10% formalin and embedded in paraffin wax. The tissues were stained with hematoxylin/eosin and examined under ×200 light microscope to assess the morphological changes. Apoptotic cells in tumor tissues were detected by In Situ Cell Death Detection Kit (Roche, Basel, Switzerland), performed according to the manufacturer′s instructions, and the photographs were observed under ×400 fluorescent microscope. Cleaved caspase-3, PCNA, VEGF, VEGFR1, VEGFR2, MMP2, and MMP9 were stained by the immunohistochemical procedure to observed the indicated protein expressions under ×400 light microscope. All of the primary and secondary antibodies were purchased from Santa Cruz Biotechnology, Inc. (Dallas, TX, USA). 

### 4.9. Gas Chromatography-Mass Spectrometry (GC-MS) Analysis of CAt Extract

GC-MS analysis was commissioned to the National Central Taiwan University Office of Research and Development’s Center for Advanced Instrumentation (Hsinchu, Taiwan). The sample was diluted using hexane (1/500), the carrier gas was helium (1 mL/min), and the injector temperature was 300 °C with an injection flow rate of 1 mL/min. Agilent 7890CB gas chromatograph (AccuTOF-GCx, Jeol, MA, USA) with an Rxi-5MS capillary column (film thickness: 30 m × 0.25 mm × 0.25 μm) was utilized to conduct GC-MS. Components were identified by comparing their mass spectra with those obtained from authentic samples or spectra of the Wiley/NIST libraries. 

### 4.10. Statistical Calculations

Results are expressed as means ± SD. Differences between groups were compared by Student’s *t*-test. Differences within groups were analyzed with the repeated measures two-way Kaplan–Meier estimator, and *p* < 0.05 was considered statistically significant.

## Figures and Tables

**Table 1:** Anti-proliferative inhibitory activities of the CAt extract in HCC and normal cells.

Cell Line	Tumor Type	CAt Extract
**Hepatocellular Carcinoma Cell**		
**HepG_2_**	Human HCC cell	27.09 ± 1.83
**Mahlavu**	Human HCC cell	33.57 ± 2.84
**Huh7**	Human HCC cell	6.09 ± 3.28
**J5**	Human HCC cell	32.83 ± 4.31
**Normal Cells**		
**SVEC**	Mouse vascular endothelial cell	68.03 ± 4.05
**MDCK**	Canine epithelial kidney cell	69.98 ± 1.56
**BNL CL.2**	Mouse liver embryonic cell	150.03 ± 9.57

Note: Values are the mean ± SD (μg/mL) at 48 h.

**Table 2:** GC-MS analysis of CAt extract.

	Compound	Percentage (%)	Chemical Structure	CAS No.	Molecular Weight
1	Thujopsene	43.36%	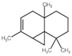	470-40-6	204.35 g/mol
2	α-Cedrene	31.67%	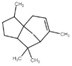	469-61-4	204.35 g/mol
3	α-Cadinene	2.73%	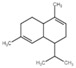	11044-40-9	204.35 g/mol
4	Cedrol	1.42%	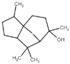	77-53-2	222.37 g/mol
5	Isolongifolene	0.52%	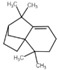	1135-66-6	204.35 g/mol

Note: GC-MS analysis was committed to National Central Taiwan University Office of Research and Development’s Center for Advanced Instrumentation (Hsinchu, Taiwan). Components were identified by comparing their mass spectra with those obtained from authentic samples or spectra of the Wiley/NIST libraries.
